# Obstinate Vomiting

**Published:** 1874-11

**Authors:** 


Enemata of Bromide of Potassium in Obstinate Vomiting.—Dr. Girabetti has obtained the very best results from the administration of enemata of bromide of potassium, in doses of from one-half to two drachms, in cases of obstinate vomiting attending the pregnant state, The same drug, also administered in enemata, has been very successful in the hands of Dr. Laborde, of Paris, in obstinate vomiting, connected with disease of the stomach, liver and intestines.—*The Medical Examiner.*

